# A retrospective analysis of survival and prognostic factors of male breast cancer from a single center

**DOI:** 10.1186/1471-2407-14-227

**Published:** 2014-03-28

**Authors:** Amr A Soliman, Adel T Denewer, Wael El-Sadda, Ali H Abdel-Aty, Basel Refky

**Affiliations:** 1 Department of Obstetrics and Gynecology, El-Shatby Maternity University Hospital, University of Alexandria, Port Said Street, El Shatby, Alexandria 21526, Egypt; 2 Department of Surgical Oncology, Mansoura Cancer Center, Mansoura 35511, Egypt; 3 Department of Nuclear Medicine, Mansoura Cancer Center, Mansoura 35511, Egypt

**Keywords:** Male breast cancer, Overall survival, Prognostic factors, Hormone receptor status

## Abstract

**Background:**

Less than 1% of all breast cancer cases are found in men, who reportedly have inferior outcomes compared with matched women patients. Ethnic differences may also affect their prognosis. Here, we investigated overall survival (OS) and major prognostic factors for male breast cancer (MBC) in a cohort of Egyptian patients.

**Methods:**

We retrospectively analyzed OS in a cohort of 69 male patients with MBC who were surgically treated at the Mansoura Cancer Center, Egypt between 2000 and 2007. We registered demographic data, age, height, weight and body mass index, tumor size, histology, number of infiltrated axillary lymph nodes, hormone receptor (HR) status and metastatic presence, and TNM staging. Patients’ OS was the primary endpoint. Patients received treatment to the medical standards at the time of their diagnosis.

**Results:**

In the 69 patients who met the inclusion criteria and had complete stored patient data, tumors ranged from T1c to T3. We could gather cancer-related survival data from only 56 patients. The collective 5-year survival in this cohort was 46.4%. Only five patients had distant metastasis at diagnosis, but they showed a null percent 5-year survival, whereas those with no lymph node infiltration showed a 100% 5-year survival. Lymph node status and tumor grading were the only prognostic factors that significantly affected OS.

**Conclusions:**

Lymph node status and tumor grade are the most important prognostic factors for overall survival of MBC in Egyptian male patients; whereas even remarkably low HR expression in MBC did not significantly affect OS. Further research is needed to understand the factors that affect this disease.

## Background

Male breast cancer (MBC) accounts for less than 1% of all breast cancer cases [1], and less than 1% of cancer incidence in male patients [2]. Prognostic factors for MBC are mostly studied in retrospective investigations with small samples. Men with breast cancer reportedly have poorer outcomes than matched women patients, even at the same disease stages, which might be because of variations in tumor biology between male and female patients [3]. Ethnic differences might also affect the prognosis of MBC [4]. As MBC is rare, knowledge about it is still limited. Here, we investigated overall survival (OS) and possible prognostic factors retrospectively in a cohort of patients of Middle Eastern ethnicity with MBC.

## Methods

### Patient selection

We retrospectively analyzed OS in 69 male patients with breast cancer who underwent operative therapy at the Surgical Oncology and the Nuclear Medicine Departments, Mansoura Cancer Center, Mansoura, Egypt, between January 1, 2000 and December 31, 2007. We surveyed medical records looking for male patients with primary diagnoses of breast cancer. This study was approved by the ethics committee of the Mansoura University, to which the Mansoura Cancer Center belongs. Each patient who met the inclusion criteria received a phone call that started with a concise informative introduction about the study and included an oral consent to take part in it, based on absolute anonymity.

### Data acquisition

We registered demographic data, age, height, weight, body mass index (BMI), tumor size, histological tumor type, number of infiltrated axillary lymph nodes (if any), hormone receptor (HR) status, presence or absence of metastasis, and TMN staging. At the time our patients were treated, human epidermal growth factor receptor-2 (HER2) status was not routinely examined in patients with breast cancer in our institution. The primary endpoint was survival of corresponding patients.

If, during the telephone interview, the patient was confirmed to have died, the relatives were asked about the exact date of death and whether the cause of death was directly related to MBC or its complications. Patients who could not be reached or who refused to give information, or for whom relatives refused to give information, for any reason were considered lost to follow-up.

Progression-free survival was omitted from this study because obtaining data on disease-free intervals is extremely difficult in the context of Egypt’s social and medical services, and was therefore extremely limited. Patients were treated by the medical standards available at the time of their diagnoses. The standard surgical therapy was modified radical mastectomy. Unfortunately, sentinel lymph node detection is not currently an established technique in our institution, nor in our country because of technical difficulties in obtaining and handling radioactive isotopes. For this reason, all patients received axillary lymph node dissection as part of their standard surgeries. They also received adjuvant chemo-, radio-, and/or hormone therapy according to available standards for female breast cancer.

### Statistics

Data was tabulated using Microsoft Excel (Microsoft Corporation, Redmond, WA, USA) and analyzed using SPSS for Microsoft Windows, version 13.0 (SPSS, Chicago, IL, USA). Breast cancer-specific OS rates were calculated by the Kaplan–Meier method. All tests assumed a 95% confidence interval (CI). *p* < 0.05 was considered statistically significant.

## Results

In the study’s time frame, we identified 69 patients who met the inclusion criteria and had complete stored patient data, from a total of 80 male patients with breast cancer. The patients’ median age was 58 years (range: 39–81 years); their mean weight was 80.30 ± 11.13 kg; mean height was 170.83 ± 4.55 cm; and mean BMI was 27.56 ± 3.99. Table 1 shows our patients’ disease characteristics in terms of tumor size, histopathological types, tumor grade, lymph node infiltration, and HR status. Most of our patients had grade 2 tumors (53.6%, n = 37). HR status was also negative in most patients (57.9%, n = 40). Tumor size had a range from T1c (n = 19), T2 (n = 48), to T3 (n = 2) (Table 2). No tumor smaller than 1 cm (stage T1c) was found in our cohort. Only five patients (8.6%) had distant metastasis at diagnosis, but 56 (77.2%) had lymph node involvement. Each patient underwent a modified radical mastectomy with axillary lymph node dissection as standard operative therapy. Subsequently, 63 patients (92.6%) received local radiation therapy and 65 patients (94.2%) received adjuvant chemotherapy. The consensus in our institution regarding adjuvant radiation and chemotherapy for these patients is ill-defined, owing to a lack of HER2 status testing and consequent treatment; absence of national recommendations, guidelines, or national follow-up programs for cancer patients; and, above all, very poor patient compliance to treatment or to the limited follow-up services available. We could gather cancer-related survival data from only 56 patients, with 10 lost to follow-up and 3 deceased as a result of non-cancer-related causes. Only 26 patients were alive at the pre-defined, 5-year, follow-up interval constituting a 5-year OS of 46.4%. At 5 years, patients who had initially presented with distant metastasis showed nil 5-year survival, whereas 100% of those with no lymph node infiltration were alive.

**Table 1:** Disease characteristics of the patient cohort

		**Number**	**Range**	**Mean ± SD**
**Tumor size (in cm)**		69	1 - 6	2.9 ± 1.1
**Histo-pathological type**	**Invasive ductal**	66		
	**undifferentiated**	3		
**Tumor grade**	**I**	14		
	**II**	37		
	**III**	17		
**Estrogen/Progesteron receptors**	**Positive**	29		
	**Negative**	40		
**Distant metastasis**	**No**	64		
	**Yes**	5		

**Table 2:** TNM classification of the patients recruited to our cohort

		**Percentage (Number)**
**T-stage**	**T1c**	27.1% (19)
	**T2**	68.5% (48)
	**T3**	3% (2)
**N-stage**	**N1**	28.6% (22)
	**N2**	25.7% (18)
	**N3**	22.9% (16)
**M-stage**	**M0**	91.4% (64)
	**M1**	8.6% (5)

**Figure 1:**
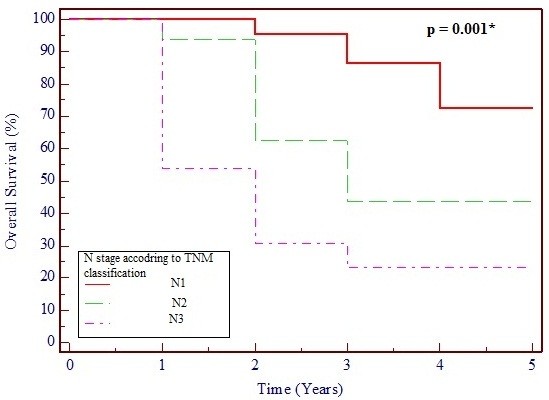
**Kaplan–Meier survival curve based on lymph node stage, showing 95% confidence interval (CI).***p* < 0.5 is considered statistically significant.

**Figure 2:**
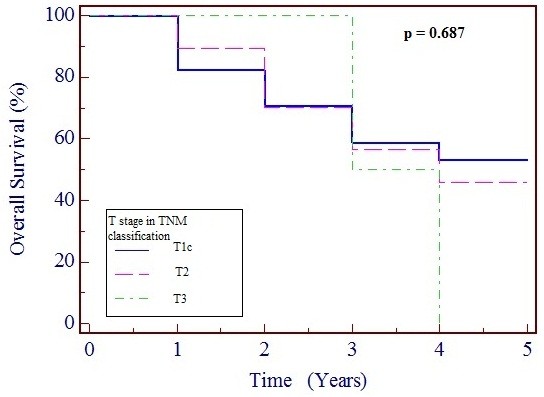
**Kaplan–Meier survival curve based on tumor stage, showing 95% confidence interval (CI).***p* < 0.5 is considered statistically significant.

Table 3 shows the effects on survival of tumor size and node involvement in terms of TNM classifications, metastasis, histopathological tumor type and grade, and HR status. The only factors that significantly affected survival were lymph node involvement (*p* = 0.001) and advanced tumor grade (*p* = 0.03), whereas tumor size (*p* = 0.687) and HR status (*p* = 0.711) had no significant effect. Figures 1 and 2 show Kaplan–Meier survival curves of TNM lymph node and tumor staging, respectively.

**Table 3:** Prognostic factors of survival in male breast cancer patients in our Egyptian cohort

		**5 year survival percentage**	**log Rank test**	**p value**
**Metastasis**	**Present**	0%	1.533	0.216
	**Not**	51%		
**Histopathological type**	**Invasive ductal**	49.1%	1.221	0.269
	**undifferentiated**	0%		
**Hormone receptor status**	**Positive**	50%	0.137	0.711
	**Negative**	42.3%		
**Tumor size**	**T1c**	52.9%	0.751	0.687
	**T2**	45.9%		
	**T3**	50%		
**Lymph node affection**	**N1**	72.7%	14.484	0.001*
	**N2**	43.8%		
	**N3**	23.1%		
**Tumor grade**	**I**	67%	10.372	0.03*
	**II**	50%		
	**III**	30%		

*p < 0.05; statistically significant.

## Discussion

In this study, the medical records of 69 Egyptian male patients with breast cancer were analyzed with regard to survival and its related possible prognostic factors. A 5-year OS rate of 46.6% can be considered low compared with published data. Giordano et al. in a cohort of 2537 men with breast cancer obtained from the National Cancer Institute’s Surveillance, Epidemiology, and End Results (SEER) program using the registry 1973–1998 found a 5-year OS rate of 63% [2]. In a Turkish cohort of 86 male patients treated over 37 years, Selcukbiricik and his co-workers reported a 65.8% 5-year OS rate [5]. In an Iranian patient cohort of 64 patients, the 5-year OS rate was 66% [6]. Moreover, O’Malley and her colleagues, in analyzing the SEER program registry between 1973 and 1997 for ethnic differences in OS of MBC, calculated 5-year OS rates of 66% for whites, 57% for blacks, and 75% for men of other races/ethnicities. Possible causes for our patients’ below-average overall survival may be the poorer quality of care provided in terms of dose calculation and application for different chemotherapeutic agents, lack of HER2 status testing and hence treatment, stereotactic planning and application of radiation, lack of a solid follow-up program for cancer patients, very poor patient compliance both to treatment and to follow-up, and finally inadequate general supportive care for cancer patients, compared with Western standards. It may also be due to late-stage diagnosis with a larger tumor burden, as all of our recruited patients had TNM stage T1c or beyond. Giordano et al. reported tumor sizes in their cohort to be 1 to <2 cm (T1c): 29.8%; 2 to <5 cm (T2): 39%; and ≥ 5 cm (T3): 5.3% [2], whereas tumor sizes in our cohort on presentation were T1c: 27.1%; T2: 68.5%; and T3: 3%. The 5-year OS rates in Giordano’s cohort by tumor size were <2 cm: 74%; 2–5 cm: 53%; and >5 cm: 37% [2]; whereas in our cohort, 5-year OS rates were T1c: 52.9%; T2: 45.9%; and T3: 50%. Our patients had worse survival than their counterparts presenting with the same tumor sizes [2] except for the two patients in our cohort who presented with T3 tumors, who had better survival rates.

We believe ethnic differences might have not played a crucial role in this case, as the data from Iran and Turkey, which both also presented data of Middle Eastern populations, had rather similar 5-year OS rates, even when compared with OS rates from developed countries.

Median age in our cohort was younger than in most other published series. Giordano et al. reported a median age of 67 years at diagnosis [2] whereas Baojiang reported a median age of 60 years in their series [7]. However, a case series of 42 Indian patients with MBC had a median age of 56 years [8]; another report of 64 Iranian patients with MBC had a mean age at diagnosis of 60.3 years [6]. Despite published data that indicated advanced age to be a predictor of worse OS [2,9], our findings did not bear this out. Although our median age of 58 years is younger than the cut-off of 65 years used in the abovementioned reports as a predictor of worse prognosis, our patients did show worse OS. This may be related to the quality of care provided, or to environmental or ethnic factors that are still unclear.

The only two prognostic factors that significantly affected survival in this cohort were lymph node status and tumor grade. Tumor grading as a negative predictor of OS seems to be controversial. Some authors found it to have a significant negative effect on OS [2] whereas others found no significant impact [5,6].

Similarly, some authors found significant negative effects on OS for tumor size [2,5], and others did not [6]. Interestingly, reports based on regional and national patient registries with large samples seem to show significant negative effects on OS by tumor size whereas single-center retrospective reports with small samples did not show such significant effects.

HR expression was present in only 42.1% of our patients, which is remarkably lower than the 65–92% seen in published series [6,8,10-14]. HR status did not significantly affect OS in our cohort, which concords with other reports where HR positivity was more highly expressed than in our patients [2,3,15]. Giordano and coworkers reported a 5.7% negative estrogen receptor status in their cohort, with a 5-year survival of 64%. HR status did not, however, significantly affect survival, as mentioned above [2]. These rates differ from what we found in our cohort where HR-negative patients were 42.1% of the cohort with a 42.3% 5-year OS in this subgroup. Lack of HER2 testing and hence treatment in our cohort may confound this absence of significant impact of HR status on survival.

This study has some drawbacks. It is a retrospective study with a small sample size. The incompleteness of the data, with 13 out of 69 patients lost to follow-up, the lack of HER2 receptor status as a standard of care, and the missing data regarding progression-free interval in the follow-up are all important flaws in this investigation.

However, this is one of a few studies of MBC in Middle Eastern men and will be of help for future research. Moreover, it throws light on the differences between prognoses and outcomes of patients in developed countries and those in developing countries, which are probably the result of differences in the quality of care between the two groups.

## Conclusions

This study showed that lymph node status and tumor grade are the most important predictors of OS for MBC in Egyptian men, and that remarkably low expression of HRs in MBC that did not have a significantly affect OS. It also indicated a lower median age of incidence of MBC in Egyptians than internationally reported data, and with still worse OS. Further research is needed into the factors that affect this disease.

## Abbreviations

BMI: Body mass index; CI: Confidence interval; HER2: Human epidermal growth factor receptor-2; HR: Hormone receptor; MBC: Male breast cancer; OS: Overall survival; SEER: The National Cancer Institute’s Surveillance, Epidemiology, and End Results program; SPSS: A software package for statistical analysis of data; TNM: An organ-specific cancer staging system adopted by the Union for International Cancer Control (UICC).

## Competing interests

The authors declare no potential conflicts of interest.

## Authors’ contributions

AS, AD, WE, AA, and BR shared in the conception and design of this study. AS, WE, AA, and BR collected data for the study and prepared it for statistical analysis. AS and BR analyzed the assembled data and interpreted it. AS, WE, and BR contributed fully to manuscript writing, and AD and AA revised the manuscript and prepared it for the final submission. All authors approved the final form of the manuscript for submission.

## Pre-publication history

The pre-publication history for this paper can be accessed here:

http://www.biomedcentral.com/1471-2407/14/227/prepub
